# Understanding Use Intention of mHealth Applications Based on the Unified Theory of Acceptance and Use of Technology 2 (UTAUT-2) Model in China

**DOI:** 10.3390/ijerph20043139

**Published:** 2023-02-10

**Authors:** Yancong Zhu, Zhenhong Zhao, Jingxian Guo, Yanna Wang, Chengwen Zhang, Jiayu Zheng, Zheng Zou, Wei Liu

**Affiliations:** 1 School of Biological Science and Medical Engineering, Beihang University, Beijing 100191, China; 2 Faculty of Psychology, Beijing Normal University, Beijing 100875, China; 3 Stanford Center at Peking University, Stanford University, Beijing 100871, China

**Keywords:** mHealth, mobile applications, user research, UTAUT-2, China

## Abstract

The COVID-19 pandemic has significantly impacted the healthcare industry, especially public health resources and resource allocation. With the change in people’s lifestyles and increased demand for medical and health care in the post-pandemic era, the Internet and home healthcare have rapidly developed. As an essential part of Internet healthcare, mobile health (mHealth) applications help to fundamentally address the lack of medical resources and meet people’s healthcare needs. In this mixed-method study, we conducted in-depth interviews with 20 users in China (mean age = 26.13, SD = 2.80, all born in China) during the pandemic, based on the unified theory of acceptance and use of technology 2 (UTAUT-2) mode, and identified four dimensions of user needs in mHealth scenarios: convenience, control, trust, and emotionality. Based on the interview results, we adjusted the independent variables, deleted the hedonic motivation and the habit, and added the perceived trust and perceived risk as the variables. Using a structural equation model (SEM), we designed the questionnaire according to the qualitative results and collected data from 371 participants (above 18 years old, 43.9% male) online to examine the interrelationships these variables. The results show that performance expectancy (β = 0.40, *p* < 0.001), effort expectancy (β = 0.40, *p* < 0.001), social influence (β = 0.14, *p* < 0.05), facilitating condition (β = 0.15, *p* < 0.001), and perceived trust (β = 0.31, *p* < 0.001) had positive effects on use intention. Perceived risk (β = −0.31, *p* < 0.001) harmed use intention, and price value (β = 0.10, *p* > 0.5) had no significant effects on use intention. Finally, we discussed design and development guidelines that can enhance user experience of mHealth applications. This research combines the actual needs and the main factors affecting the use intention of users, solves the problems of low satisfaction of user experience, and provides better strategic suggestions for developing mHealth applications in the future.

## 1. Introduction

During the COVID-19 pandemic, traditional healthcare faced many problems, such as a significant increase in the number of people attending medical appointments, longer waiting times, and a shortage of medical and nursing staff [1,2]. At the same time, traditional medical and healthcare services are restricted by geographical and economic factors, making it increasingly difficult for patients to see a doctor and increasing the burden that people must bear in pursuit of quality services [3,4,5]. In addition, medical disputes due to asymmetric information about the pandemic occur from time to time, and the tension between doctors and patients even affects overall social harmony [6,7]. Traditional medical clinics can hardly provide people with timely and efficient medical services and a satisfactory consultation experience, which cannot meet people’s growing rigid demand and seriously affects the development of the “Healthy China” strategy [8].

With the rapid development of the Internet and related technologies, Internet healthcare is becoming an effective means to solve traditional healthcare’s problems in the post-pandemic era [9,10]. Internet healthcare has broken through time and space restrictions. It becomes a bridge of communication between patients and doctors, realizing inter-temporal consultation and advice, allowing patients to talk to doctors about their conditions and physical status [11]. Doctors make basic judgments and guidance based on patients’ critical medical information, effectively improving the efficiency of medical resource utilization. Catalyzed by the rapid development of mobile Internet and the popularization of smartphones, mobile healthcare (mHealth) applications, an indispensable and critical component of Internet healthcare, are emerging globally [12,13,14]. In China, various innovative mHealth applications have attracted wider attention for making healthcare services more convenient and proved to be an effective means to solve persistent problems in the domestic healthcare system (e.g., shortage of resources and tension between doctors and patients).

However, there are many issues with the current mHealth applications, such as a lack of innovation, homogenization, poor user experience (UX), lack of user trust, and low user stickiness [15,16]. The research of the mHealth applications in facilitating self-management has just focused on patient experiences involving a single chronic condition [15]. Users experience technical difficulties with their smartphones when they upload readings from their meters to their mobile phones, and the evaluation feedback system is the same [16]. Qualitative studies linking user characteristics to favorable UX have been underrepresented in the literature [16]. To improve UX of mHealth consultation, it is urgent to focus on the users themselves, combine the main factors such as use intention and user demand, and provide better product designs. We aim to solve the above issues by focusing on the users, combining the main factors such as use intention and user needs, and providing better product design suggestions for developers. As an emerging industry, mHealth is based on the mobile Internet and uses mobile devices as a carrier to provide healthcare services and information to patients through mobile applications. According to the difference of the main types of services provided, mHealth applications can be divided into five categories [17,18,19]: (1) health management, mainly to provide users with health management services; (2) medical consultation, mainly to build an online communication platform between users and doctors so that users can seek medical consultation remotely online; (3) medical supporting platform, mainly to provide users with auxiliary process services to improve the efficiency of offline medical treatment; (4) doctors’ tools, mainly provide medical-related information or help with patient management for doctors and other professionals to improve their work efficiency; (5) medical e-commerce, mainly provides users with medical supplies and services for purchase.

The mHealth system started to be built earlier in China, and the number of existing programs has exceeded one hundred thousand [20]. Previous research mainly focused on mHealth technologies and services. In terms of mHealth technology, there are problems in its technical and service aspects at the early stage of development. In terms of information technology (IT), device’s issues of compatibility and connectivity have seriously hindered UX. In terms of service, the coverage is small, mainly in diabetes and mental health. In addition, researchers pointed out that although the cost of healthcare services can be reduced and the efficiency of diagnosis can be improved through mHealth, it brings new issues, e.g., privacy leakage. Kayyali et al. (2017) surveyed the current situation of user awareness of mHealth applications [21]. They found that public awareness of mHealth applications is low, and the usability is not as good as expected. With the development of IT [22], these applications have gradually overcome the compatibility issues of mobile devices, and the issues in terms of functionality and services have been significantly improved.

In recent years, the number of mHealth users has surged due to the outbreak of the pandemic, and the consequent new issues have provided a new focus for related research. Pires et al. (2020) classified the functions of mHealth applications into seven types [23]: literature, patient monitoring, diagnosis, personal care, psychological health, educational applications, and social networking applications. Based on the study of the current applications included in each category, they suggested four limitations [23]: usability, ethics, network, and management.

In terms of research on the use intention to mHealth, Zapata et al. (2015) demonstrated that mHealth applications have a significant impact on usability to adapt to user needs [24]. It had a positive effect on adoption intention, while resource limitation had a reverse impact on adoption intention. Peng et al. (2016) explored public perceptions of mHealth applications through a qualitative study, providing suggestions for developing and evaluating these applications from a UX perspective [25]. They identified privacy and security concerns, user trust, product credibility, and accuracy as the issues. A study by Bhuyan et al. (2017) showed that privacy and security concerns become a hindrance for users in mHealth scenarios [26].

In summary, recent research works are mainly based on analyzing these applications from political, economic, social, and technological perspectives without being able to clarify the shortcomings and issues from the essential user needs. This leads to the development of future products deviating from UX. In addition, many studies use a single theoretical model as the basis or select individual factors for conducting research, resulting in less comprehensive analysis of the influencing factors.

The unified theory of acceptance and use of technology (UTAUT) model explains the factors that influence the acceptance and use of technology by individual users and is widely used to study the intention to use a product [27,28,29]. The model has four key constructs: performance expectancy (PE), effort expectancy (EE), social influence (SI), and facilitating condition (FC). It also applies gender, age, experience, and voluntariness of use, posited to moderate the impact of the four key constructs on use intention and behavior. It reports accounting for 70% of the variance [30]. This model has been successfully applied to technological innovation and its diffusion in various fields, covering areas such as information systems, marketing, social psychology, and management. The unified theory of acceptance and use of the technology 2 (UTAUT-2) model is a modified version of UTAUT, which allows the model to be applied to a broader range of people (i.e., users, consumers, and customers), thus achieving a higher degree of explanation of behavioral intention (BI) [31,32,33]. This modified model retains all four core variables in UTAUT, removes voluntariness from the moderating effect, and adds three core variables: price value, habit, and hedonic motivation.

This model is now widely used in the mobile Internet industry. Slade et al. (2014) expanded on UTAUT-2 with five variables, self-efficacy, innovativeness, trialability, perceived risk (PR), and perceived trust (PT), based on the latest research on mobile payments [34]. These variables were used to examine the user behavior (UB) and verify the applicability of UTAUT-2. Oechslein et al. (2014) introduced three characteristics of users’ social networks, personal information, and reading behavior into UTAUT-2 [35]. They tested this model on social recommendation systems by trying it on 266 students. Arain et al. (2019) considered the shortcomings of this model [36]. They introduced five core variables, ubiquity, information quality, system quality, appearance quality, and satisfaction, thus expanding the research area covered by this model and enabling it to support the exploration of technology acceptance and UB. Alalwan et al. (2017) conducted a study on takeaway ordering mobile applications and proposed an extended model by combining UTAUT-2 and the functionality of takeaway ordering [37]. The analytical results of this study show that this model effectively predicts users’ satisfaction and intention. Research conducted by analyzing mobile phone technology and mobile government services in Saudi Arabia found that UTAUT-2 could be modified and extended by considering new structures suitable for adoption by Arab customers. Research conducted by analyzing mobile phone technology and mobile government services in Saudi Arabia found that UTAUT-2 could be modified and extended by considering new structures applicable to the context of adoption by Arab customers [38].

Thus, UTAUT-2 is a relatively mature model with high predictive validity. It provides strong theoretical support for the study of factors affecting user acceptance behavior of products in various fields in mobile technology-related research. At the same time, in the practical examination, the introduction of new and appropriate variables to modify this model according to the actual situation can promote more substantial explanatory power in specific technical procedures. Therefore, this study selected UTAUT-2 as a theoretical basis for an in-depth understanding of the key factors that affect user intention to experience mHealth applications.

By combining qualitative and quantitative user research methods, this study provides insights into the factors that influence user needs to use mHealth applications. By further improving the research on product design strategies, providing a theoretical basis and strategic support for the design and development of related products, helping to increase use intention, improving product usability and satisfaction, and promoting the use of mHealth applications, we aim to improve product usability and pleasure and promote the popularity and development of mHealth applications.

## 2. Materials and Methods

The user research combined both user interviews and questionnaires. First, we conducted user interviews to gain an in-depth understanding of users’ behaviors, feelings, and expectations when using mHealth applications and to extract users’ primary needs in mobile scenarios. Secondly, based on UTAUT-2, we designed a research questionnaire on user acceptance behaviors. We explored the key factors influencing use intention of mHealth applications. The results of the user research phase were used for the mHealth application design strategy. They provided the data and theoretical basis for the subsequent research.

### 2.1. User Interviews and Qualitative Analysis

The purpose was to collect users’ behaviors, feelings, and expectations by conducting one-on-one interviews with users using mHealth applications. Then we transcribed, coded, and analyzed the interview results based on grounded theory to summarize the primary user needs and scenarios [39,40,41,42].

#### 2.1.1. Participants and Procedure

In this research, we determined the interview participants by the snowball sampling method. First, we interviewed an IT manager and a teacher who often teaches online, and then they recommended participants who were considered eligible for this research. Based on the UTAUT-2 model, the final participants were determined according to their age, gender, and Internet experience as screening criteria. We recruited twenty 18–40 years old participants (eleven females and nine males. M age = 26.13 s, SD = 2.80). All of them were cisgender and used mobile phones for more than 5 h per day. After confirming the eligibility, all participants provided informed written consent for the study protocol as approved by Institutional Review Board (IRB) at our institute. These interviews were conducted entirely online due to the COVID-19 limitations.

We conducted the interview. Because of COVID-19, we conducted the interview online. The interview outlines were semi-structured. UTAUT-2 has independent variables: PE, EE, SI, FC, HM, PV, and habit. It also introduces age, gender, and experience (Exp) as moderators between the explanatory variables and BI [27]. The purpose was to adjust and re-describe the meaning of the variables through interviews and qualitative analysis. The questions were divided into three parts: (1) understanding the participants’ basic information, including the age and gender, and guiding them into scenarios; (2) understanding user needs and pain points from the conventional healthcare experience; and (3) understanding expectancy, experiences, and behavioral preference when using mHealth applications in healthcare scenarios. At the end of the interviews, we imported all interview data into the Nvivo11 software (qsrinternational.com/nvivo-qualitative-data-analysis-software, accessed on 1 February 2023), drawing on the grounded theory. A systematic process was used to summarize and code the interview data, and then a three-level coding process was used to distill the core user needs for mHealth applications. The question guide is shown in Table 1.

#### 2.1.2. Data Analysis

The purpose of the qualitative analysis was to adjust the model variables and formulate hypotheses based on the new variables afterward. In addition, the questionnaire was designed based on the findings of the qualitative analysis. In this study, the data were analyzed based on the variables in the UTAUT-2 model using the grounded theory qualitative research method [43]. The operational process was mainly through a three-level coding procedure, including selective coding, axial coding, and open coding [44,45]. To avoid subjectivity in coding, we drew on consensus coding to code the textual material. A total of two or more coders were required to work together in the coding process [46]. Two research team members conducted the coding phase of this study, and each coding process required a discussion and consensus before a preliminary coding could be formed.

First, we normalized and labeled the interview transcribed material with textual data for open coding. After the interviews were transcribed, we organized the material related to the UTAUT-2 model and named it in the context of the interviews. For example, “I think it is not difficult to use an application to see a doctor. As long as I understand it, I can use it” and “I think the effect of offline online treatment is similar, and I think online is more convenient”. We labeled this part of the material as “low learning cost” and “mHealth is more convenient”. In the second step of axial coding, we combined the content of the open coding with the variables in the UTAUT-2 model and the frequency of occurrence to establish that relationships, such as “low learning cost” and “convenient service”, are related to the FC in the model, which is similar relationships. In this process, if the interviewed material appears inconsistent with the current classification, new dimensions are generated until no new dimensions are generated; then theoretical saturation is considered reached. Finally, selective coding was performed, in which we linked with more generalized categories. This part of the coding is the basis for the model’s adjustment (removal and redefinition) of variables. For example, after the three-level coding, we readjusted the definition of the FC: the perceived ease-of-use available during the use of the product and the degree of technical support of the system. For example, in the interviews, users mentioned two types of PT-related needs (i.e., “need to refer to evaluation information” and “need to have professional assurance in mobile health care”) and aggregated these two conceptual categories into “trust”. Therefore, PT was added as a new independent variable in UTAUT for the study. The core categories are shown in Table 2.

#### 2.1.3. Adjusting and Redefining Variables

The variables were based on UTAUT-2. Still, the interviews revealed that some of the variables in the model did not fit into the mHealth scenarios and had no effect on use intention to accept such applications. Therefore, we adjusted the variables and redefined core variables to make the model more compatible with our scenarios.

- The hedonic motivation and habit variables were removed. Through the interviews, we found that most of the scenarios were focused on the medical care context, where users consider factors such as access, efficiency, and quality of medical services. Hedonic factors were not part of the user needs in this context. In addition, medical-related needs were relatively low-frequency in their daily life, and mHealth did not form a habit.
- The PR variable was added. Chang et al. (2016) argued that users were uncertain about the outcome of their shopping behaviors, which even harms users [47]. PR refers to the uncertainty of the development of behavior process. Medical services are related to people’s life and health, so controlling medical risks has always been a concern. We found the participants chose different ways of accessing medical and health care services, depending on the severity of the disease. When they were faced with more severe conditions, they would be more cautious in deciding whether to use mHealth applications or not.
- The PT variable was added. With the development of socioeconomic, trust has become one of the important influencing factors in the transaction process, affecting use intention and behavior. In a study on user acceptance behavior of e-commerce, Pavlou (2003) introduced trust variables and found that trust enhanced use intention [48]. We found when the participants choose online consultation platforms or doctors to consult, they actively paid attention to the background information, such as user ratings and doctor’s titles, and then they determined the level of trust in the information and services provided by mHealth applications. The level of trust affects the subsequent use intention.
- The experience variable was removed. As the development of emerging technologies, there is no significant difference in UX of other mobile applications.

To accurately measure the dimensions of each variable, we redefined the variables based on the specific context of mHealth: (1) PE refers to the user’s belief that the efficiency and quality of healthcare services can be improved when using the application. It is reflected in the user’s ability to access basic healthcare information or services anytime, anywhere, conveniently, and quickly through mobile devices. (2) EE refers to the degree of difficulty users feel in using the application. It is reflected in the learning cost and effort users need to put in. (3) SI refers to the influence of other people’s behavior and attitude or the surrounding environment on the use intention, mainly including the recommendation of friends and relatives and the pressure felt by the media network in the environment. (4) FC refers to the user’s perception of convenience and the degree of technical support, specifically the ability to receive timely help and support. (5) PV refers to the user’s perception of the price of the service. This includes the willingness to pay for some of the features or services and satisfaction with the information or services received. (6) PR refers to the user’s expectation of the impact of uncertainties or losses. This is expressed as the assessment of possible losses in terms of health, property, and privacy. (7) PT refers to users’ expectation of the degree of trust based on their understanding of brand, doctors, and professionalism. (8) BI refers to the user’s tendency to use or recommend an application. (9) UB refers to the user’s activities such as recognition and active recommendation.

#### 2.1.4. Formulate Hypotheses

After adjusting and redefining the variables in the model, we proposed the following nine hypotheses for the correlations of the variables in the mHealth scenarios, as shown in Table 3 below.

For the nine hypothetical latent variables, we set the observed variables for each latent variable, as shown in Table 4.

**Figure 1:**
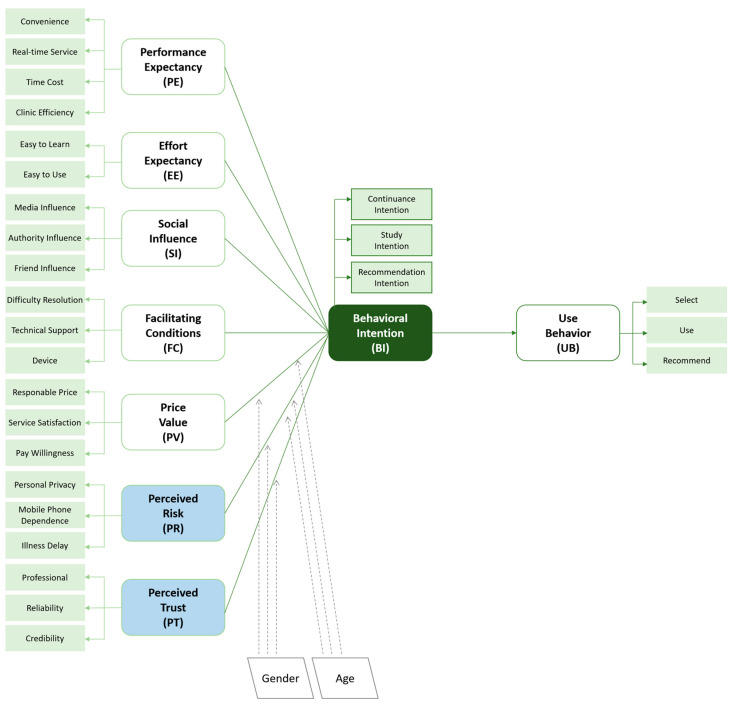
The adjusted UTAUT-2 model based on the interviews.

The adjusted user acceptance behavior model is shown in Figure 1.

### 2.2. Questionnaires

The questionnaire was designed based on the qualitative results and the hypotheses. The purpose was intended to examine the interrelationships among the various mHealth adoption factors. After developing the questionnaire, we had a pilot test using psychological experts and college students to ensure that the respondents can fully understand the questions. We distributed 30 questionnaires with a few items rephrased.

The body of the questionnaire consisted of two parts: (1) basic information, including gender, age, education level, functions used, and disease type; (2) the core measurement was designed with the variables in Figure 1 with no less than three measurement items for each variable, adapted from UTAUT-2, to ensure the validity and reliability of results. The options for each question ranged from “strongly disagree” to “strongly agree”, corresponding to a score of 1 to 5.

Data were collected through a professional questionnaire platform (wjx.cn, accessed on 1 February 2023), and the respondents were recruited online. We set screening questions in the basic information section to determine the respondents. The information on gender, age, and education were not used as screening conditions to ensure that the characteristics of the user group were reflected as honestly as possible. The screening criteria were older than 18 years old (i.e., in China, many students under 18 years old are in school and cannot use mobile phones for a long time during the day) and whether they were concerned about mHealth. Finally, 430 questionnaires were distributed, and 371 valid data were collected and analyzed.

### 2.3. Statistical Analysis

The data were analyzed using SPSS and AMOS (ibm.com/products/structural-equation-modeling-sem, accessed on 1 February 2023). Through the descriptive statistical analysis of each item the questionnaire, the average value, standard deviation, skewness and kurtosis of each item were obtained. The standard deviation distribution range of the scale was between 1.025 and 1.378, and the numerical fluctuation was small, relatively close to the average level. The peak and skewness of the sample data were consistent with the normal distribution standard proposed by Kline [49], which can be accepted as normal distribution. Therefore, the sample data were suitable for further analysis. Descriptive analyses were conducted with means and standard deviations for continuous variables. Models were estimated using the maximum likelihood method. The statistical significance value was taken as *p* < 0.05.

## 3. Results

### 3.1. Findings of the Qualitative Study

The results of the three-level coding are shown in Table 5, showing the user needs and pain points in the mHealth scenarios. Based on the grounded theory, we summarized four dimensions of user needs: convenience, control, trust, and emotionality.

### 3.2. Participants of the Quantitative Study

As shown in Table 6, a total of 371 participants were recruited. In terms of gender, the percentage of participants was slightly higher among women (56.1%) than among men (43.9%), reflecting more women than men were users of mHealth applications. In terms of age, there were far more users aged 18–25 (42.0%) and 26–30 (34.2%) than in other age groups. These two age groups were the main active user groups on the Internet, indicating that the young people were the primary users of these applications. Their education level was mainly concentrated in bachelor’s (40.4%) and master’s (43.1%) degrees, and the number of other education levels was low. This indicated that the education level of this questionnaire study was relatively high.

The usage and disease types are also described in Table 4. The number of people who used appointment booking and information search services was higher, accounting for 65.8% and 61.2%, respectively. This indicated that users preferred to search information and book offline appointments on the Internet when they have medical needs. The number of people choosing consultation and medical services was lower, accounting for 49.1% and 47.4%, respectively. This indicates that the acceptance of choosing to seek medical consultation online was not high. Reading information and other functions accounted for 31.5% and 8.9%, respectively, indicating that they were not the core needs. In terms of specific diseases, many respondents used mHealth applications for minor illnesses, accounting for 71.7%. This was followed by health monitoring and chronic disease, accounting for 11.9% and 11.3%, respectively. The proportion of acute and severe illnesses and post-illness rehabilitation was only 5.1%. This indicated that the procedure was more suitable for relatively non-urgent scenarios, such as minor illness, while for acute and severe illness, in person visits were more appropriate.

### 3.3. Reliability and Validity Analysis

In the statistical analysis of the data, we used PE, EE, SI, FC, PV, PR, PT, BI, and UB as latent variables. We used the measurement items under each latent variable as observed variables. Table 7 reports internal consistency measures Cronbach’s Alpha (α) for all items of the scales, ranging from 0.83 to 0.90. Thus, the reliability of this scale was good. For convergent validity, we required the loading of each standardized factor to be greater than 0.7, each variable’s composite reliability (CR) to be greater than 0.7, and the average variance extracted (AVE) to be greater than 0.5. The standardized factor loading, CR, and AVE all met the above requirements. Therefore, we can conclude that the scale in this questionnaire had good convergent validity.

The mean value (M), standard deviation (SD), skewness, and kurtosis of each item were obtained by descriptive statistical analysis in the scale section, as shown in Table 5. The SD of the scale ranged from 1.025 to 1.378, and the values fluctuated less and were closer to the average. The skewness and kurtosis of the sample data met the normal distribution criteria [50,51].

The mean value of each question item in PE, EE, and SI was higher than 3.5 points, indicating that users rated the program highly in terms of efficiency, ease of use, and influence. Regarding FC, the mean score for FC1 was below 3.5 (M = 3.30, SD = 1.284), indicating that users currently had a low acceptance of receiving timely guidance while using the application. This indicated that users still needed to acquire more timely and accurate advice when they encountered problems operating the applications. Regarding price value, PV4 was below 3.5 points (M = 3.47, SD = 1.208), indicating that users had low confidence in the value aspect. The ratings differed significantly from those of the other variable items in terms of PR. All items were below 3 points. Most users currently had a low level of PR in using mHealth applications. They did not excessively worry about risk issues such as privacy leakage. Regarding PT, the average score for each item ranged from 3 to 3.5. This indicated that users had some trust in the authority and professionalism of the service. However, its trustworthiness still needed to be improved. Regarding BI and UB, the items BI2, BI4, UB2, and UB3 were all in the range of 3–3.5 points. We found that use intention to recommend the applications was relatively low, and at the same time, mHealth was not the preferred way for some users.

### 3.4. Inter-Variable Relationships

Based on the above questionnaire study and related hypotheses, in this user acceptance behavior model, we used the seven variables PE, EE, SI, FC, PV, PR, and PT as exogenous latent variables and BI and UB as endogenous latent variables. By establishing a structural equation modeling (SEM) based on UTAUT-2 [52], we determined the interrelationships among the variables and tested the hypotheses.

#### 3.4.1. Fitting Degree of the Model

Before performing path analysis on SEM, we evaluated its fit degree to describe the correlation between the pre-defined model and the actual data. The fit index included χ2/df, root mean square error of approximation (RMSEA), goodness-of-fit index (GFI), comparative fit index (CFI), incremental fit index (IFI), Tucker–Lewis index (TLI), etc. When the fit values reach the recommended standard values, the model has good explanatory and predictive power and can be used for further analytical studies. Table 8 shows the fit results of this SEM. χ2/df, RMSEA, IFI, TLI, and CFI values [53] of the SEM all reached the recommended standard value. GFI, adjusted goodness-of-fit index (AGFI), and normed fit index (NFI) did not meet the recommended standard value but were close to the value. Baumgartner and Homburg (1996) have pointed out that the model’s fit is affected by various factors [54]. Thus, researchers should not arbitrarily adjust the model to obtain a higher fit but evaluate it according to the actual situation. The present model was based on UTAUT-2 and had strong explanatory power. Thus, we argue that this model has met the acceptable fit value.

#### 3.4.2. Path Analysis of the Model

Path analysis was used to investigate the influence relationships between variables of the SEM and test the model’s hypotheses. If the influence relationship between paths was significant, the hypothesis of the path was valid, and vice versa. Table 9 shows the detailed path coefficient and significance of each variable. The variables influencing use intention included PE, EE, SI, FC, PR, and PT, while the influence of PV was insignificant. Among these variables, the path coefficient of PR was negative, while the rest were positive. PR negatively hindered use intention, and the rest had positive facilitating effects. UB has a positive effect as well. Therefore, hypotheses H1, H2, H3, H4, H6, H7, and H8 held, while hypothesis H5 did not hold.

#### 3.4.3. Analysis of the Moderating Effect of Age and Gender

In this study, the independent variables were all latent variables and can be considered as continuous variables. The moderating variables included gender and age, both of which were categorical variables. A significant difference in the path coefficient meant that the moderating variable had a significant effect on that path. In this study, gender and age were used as moderating variables. For the gender variable, we established two groups divided into male and female. For the age variable, the primary user group was selected and divided into 18–25 and 26–30 years old. We analyzed the models after restricting each regression coefficient to be equal. We found that neither of the models with equal path coefficients for gender nor age reached statistical significance (*p* = 0.074, *p* = 0.628) with the models without restricting the path coefficients. Therefore, we concluded that neither gender nor age played a moderating role in this model.

## 4. Discussion

This study aimed to explore the factors that enhance user experience and intention to use when users use mHealth applications. Using structural equation modeling, we found that six factors, PE, EE, SI, FC, PR, and PT, were the main factors that impacted users’ intention to use the sample.

PE positively affected use intention, indicating that mHealth applications can help users improve the efficiency and effectiveness of medical treatment and help improve their use intention. PE was reflected in medical consultation efficiency, flexibility, and usefulness. Integrating online and offline services and operating them online as much as possible reduced users’ offline queuing time, broke the restrictions of time and location, and improved overall PE of the applications. EE positively affected use intention, indicating that users felt it easy to understand process of usage. Enhancing EE can be achieved by improving the ease of learning, ease of use, and operability. By simplifying the interaction process of online registration, consultation, and medication purchase operations and showing users a clear and straightforward interface design, users can quickly learn to use mHealth applications. Enhancing users’ EE further enhanced their use intention. FC positively affected use intention. FC was divided into internal and external contributing factors. External contributing factors depended on network conditions, device support, etc. Internal contributing factors were timely help and support, such as user interface design guidelines. When the FC was better in terms of convenience, the use intention was more substantial. We can improve convenience and increase use intention by setting up proper guidance and help support.

There was no significant effect relationship between PV and use intention. Thus, it cannot prove that the low price of Internet healthcare services improved use intention. The reasons were as follows: (1) the price required for consultation service, registration service, and medicine purchase service provided by mHealth applications was not much different from that required offline. There was no significant price advantage, and users did not have a strong perception of price; (2) mHealth scenarios were often accompanied by people’s fear of health threats. Some people were willing to pay a particular price to obtain adequate treatment and were not overly concerned about the price, such as registration, consultation, and medicine purchase. Therefore, there was still room for improvement to enhance use intention by improving the value perceived by users.

SI positively affected use intention, indicating that the more social groups positively influenced more users in their surroundings, the stronger their use intention. The results corroborate other researchers’ findings [55]. In their study, perceived usefulness (PU), attitude (ATT), perceived ease of use (PEOU), and BI influenced SI, and society often plays a crucial role in convincing a user to adopt mHealth services. In addition, to online and offline media promotion, we also increased users’ positive message reach a higher rate through social information flow and other means to promote SI and enhance use intention. PT positively affected use intention, indicating that when users’ trust in the applications increased, use intention increased. Through the interviews, we found that users would actively focus on the platform’s brand authority and the doctors’ professionalism to judge whether they can obtain the ideal information and services. mHealth platforms should build a good brand image, strictly supervise the resident physicians, and ensure the quality of medical services.

In the service process, users should be provided with accurate and reliable professional information to enhance their trust and thus enhance use intention. Users’ sense of trust and control is satisfied when they perceive that the services provided by mHealth applications are reliable and meet their expectations [56,57]. Conversely, users would need better references. PR negatively affected use intention, indicating that the higher the user perceived the risk of using the applications, the lower the use intention. This finding is consistent with another related study [55] that found users’ anxiety is mainly about technology. Technology anxiety may be from a lack of understanding of technology and fear that there is a risk of a privacy breach. In mHealth scenarios of our study, the risk was always one of the essential concerns for users. Risks included privacy leakage, medication, fraud, etc. The level of risk perceived was higher than expected, which prevented users from continuing to use the applications or even leading to abandonment. We can continue reducing risk concerns and improving use intention by enhancing features and regulations. Therefore, when designing and developing these applications to enhance positive SI and PT and lower PR, we should highlight professionalism, authenticity, and reliability in four aspects: emphasizing user feedback, comparing service metrics, visualizing service status, and communicating safety and security.

There was no significant effect of gender and age on this model, indicating that gender and age, as moderating factors, had insignificant moderating effects on use intention and behavior. In addition, this study did not set more moderating variables due to time and condition constraints. The possible moderating effects of other factors, such as health and economic status, on this model cannot be excluded.

Although mHealth applications have met some user needs in medical scenarios, users’ perception of FC is still weak, while lacking a sense of emotion negatively affects use intention. When designing and developing mHealth applications, we should focus on bringing users an emotionally pleasant experience by improving the contributing factors to enhance use intention. For example, the design and development should improve the matching between information and users [58,59,60]. mHealth applications should be designed to improve the quality of information that matches the users, based on their interests and information, and delivers professional and authoritative content. The design should create kinship-related scenarios because there is a strong kinship relatedness among the mHealth user group. Therefore, we should give more convenience to such scenarios to enhance the kinship connection and create a good emotional experience.

This study is limited by time, geography, and resources. First, this study was conducted in China. Still, the quantitative analysis did not restrict where the cities were located, so the findings need to properly reflect differences in population distribution and users across cities. Second, we limited the selection of study participants to those with experience using mHealth applications and did not study those without experience. Third, we should have differentiated their health status at the time of participation, so there may be differences in the mindset and needs of healthy people and patients. In addition, previous study has shown that gender differences have a moderating role in social influence and behavioral intentions [54]. In this study, gender differences were restricted to males and females only, and the findings did not reflect the effects of gender differences. Our study variables involved only those in the UTAUT-2 model. Educational factors, personal attitudes, and technology anxiety were not studied. Follow-up work can continue to iterate the model, expand the distribution of the sample of subjects in more dimensions, enhance the sample’s representativeness, and verify the influence of usage experience, health status, and other aspects on user needs and use intention.

## 5. Conclusions

Technology is a necessary tool in the digital age, and this is also true in healthcare. mHealth applications can act as a medium for initial disease prevention to create a connection between patients and physicians. Both qualitative and quantitative studies have shown that improving UX in PE, EE, SI, FC, and PT and reducing PR at the time of use can effectively increase use intention. The findings of this study provide theoretical support for the subsequent functional and interface design of mHealth applications and the better promotion of this technology.

## Figures and Tables

**Table 1:** The interview question guide.

	Question	Purpose
Basic information	Age, occupation,	To obtain basic information and guide participants to understand the interview situation
	and recent physical condition	
Conventional healthcare scenarios	Recent or impressive offline medical experience	To understand participants’ needs and pain-points in conventional healthcare scenarios
	Reasons for choosing offline medical treatment	
	Problems encountered in offline medical treatment	
mHealth scenarios	Which mHealth applications have the participants used?	To understanding expectancy, experiences, and behavioral preference
	How do the participants gain access to mHealth applications?	
	What functions have the participants used? How about the UX?	
	How complicated is a mHealth application to use?	
	What problems do the participants encounter when using mHealth applications?	
	What should the participants consider when choosing a doctor?	
	What is the attitude toward the privacy protection?	
	Do the participants recommend mHealth applications?	
	What are the functional expectations?	

**Table 2:** Examples of qualitative analysis: PT.

Selective Coding	Axial Coding	Open Coding	Example Quote
PT	Need: Evaluation information	Referrals	“*I saw that someone recommended it, and then quite a few people forwarded it, which would feel more appropriate for use at the time*.”
		Refer to doctor information	“*There are doctors online whose information is consistent with offline, but it could be more transparent. Offline registration, even if the information is simple, will increase trust*.”
	Need: Mobile health care with professional guarantee	The platform is professional and trustworthy	“*Much health knowledge is available on the general application, but it is better than the specialized medical application. I still trust its professionalism more*.”

**Table 3:** Correlated hypothesis of each variable.

Number	Hypothesis
Hypothesis 1 (H1)	*PE has a positive effect on use intention to use mHealth applications*
Hypothesis 2 (H2)	*EE has positive effects on use intention*
Hypothesis 3 (H3)	*SI has positive effects on use intention*
Hypothesis 4 (H4)	*FC has positive effects on use intention*
Hypothesis 5 (H5)	*PV has positive effects on use intention*
Hypothesis 6 (H6)	*PR harms use intention*
Hypothesis 7 (H7)	*PT has positive effects on use intention*
Hypothesis 8 (H8)	*Behavioral Intention (BI) has positive effects on UB*
Hypothesis 9 (H9)	*The user’s gender and age have an impact on the overall model*

**Table 4:** Definition of latent and observed variables.

Latent Variable	Observed Variable	Content of Variable
Performance Expectancy (PE)	PE1	Convenience
	PE2	Real-time service
	PE3	Time cost
	PE4	Efficiency of visit
Effort Expectancy (EE)	EE1	Easy to learn
	EE2	Easy to learn
	EE3	Easy to use
	EE4	Easy to use
Social Influence (SI)	SI1	The influence of friends
	SI2	The influence of media
	SI3	The influence of authority
	SI4	The influence of friends
Facilitating Condition (FC)	FC1	Troubleshooting
	FC2	Technical support
	FC3	Equipment condition
Price Value (PV)	PV1	Price paid
	PV2	Service satisfaction
	PV3	Service satisfaction
	PV4	Willingness to pay
Perceived Risk (PR)	PR1	Privacy issue
	PR2	Dependency of mobile device
	PR3	Dependency of mobile device
	PR4	Delayed illness
Perceived Trust (PT)	PT1	Professionalism
	PT2	Trustworthiness
	PT3	Reliability
Behavioral Intention (BI)	BI1	Intention to continue using
	BI2	Intention to continue using
	BI3	Intention to learn
	BI4	Intention to recommend
User Behavior (UB)	UB1	Selection
	UB2	Use
	UB3	Recommendation

**Table 5:** The three-level coding.

Theme	Sub-Theme	Paraphrase/Description
Convenience	Immediate medical response	Not limited by time and place
		Do not like to plan ahead
		Use only when needed
	Saving time and be easy to use	Low learning cost
		Long waiting in using affect UX
		mHealth is more convenient
Control	Reducing the risk of consultation	Worry about incomplete communication with doctor
		Choosing consultation method according to the illness
		Uncertainty about whether the doctor is in person
	Protecting private information Making autonomous decision	Do not mind privacy leakage
		Used to privacy disclosure
		Would not take doctor’s advice
		Independent search for relevant information
Trust	Referencing evaluation information	Referrals
		Referring to doctor information
	Professional coverage for mHealth	mHealth should be authoritative and professional
		Seeing doctors in person is more reliable
Emotionality	Consultation results with relevance	Homogenization of consultation results (individualized guidance is weak)
		Results are somewhat helpful
		Online consultation is not worth paying for
	Doctor‘s good service and bedside manner Seeing interest content	Doctor‘s bedside manner is good
		Doctor’s response speed affects UX
		Do not want to see irrelevant information
		Online consultation is not customized
		Need personalized medical information

**Table 6:** Descriptive statistics of demographic variable, application usage, and disease type (n = 371).

Statistical Variables		n	%
Gender	Male	163	43.9
	Female	208	56.1
Age	Under 18	32	8.6
	18–25	156	42.0
	26–30	127	34.2
	31–40	43	11.6
	Above 40	13	3.5
Education Level	Highschool	22	5.9
	Bachelor’s	173	46.7
	Master’s	160	43.1
	PhD	16	4.3
Application Usage	Consultation	182	49.1
	Medical service	176	47.4
	Appointment booking	244	65.8
	Search information	227	61.2
	Read information	117	31.5
	Other	33	8.9
Disease Type	Minor illness	266	71.7
	Chronic	42	11.3
	Acute and severe illness	11	3.0
	Post-illness rehabilitation	8	2.1
	Health monitoring	44	11.9

**Table 7:** Results of reliability and validity.

Latent Variable	Observed Variable	Standard Factor Loading	Cronbach’s α	CR	AVE	M	SD	Skewness	Kurtosis
PE	PE1	0.81	0.86	0.86	0.60	3.73	1.137	−0.955	0.228
	PE2	0.78				3.77	1.182	−0.891	−0.062
	PE3	0.76				3.95	1.127	−1.139	0.559
	PE4	0.75				3.74	1.143	−0.882	0.033
EE	EE1	0.79	0.89	0.89	0.66	3.78	1.144	−1.001	0.313
	EE2	0.83				3.85	1.137	−1.038	0.392
	EE3	0.82				3.84	1.025	−0.908	0.455
	EE4	0.81				3.82	1.065	−0.982	0.537
SI	SI1	0.81	0.86	0.86	0.60	3.87	1.132	−1.026	0.321
	SI2	0.82				3.78	1.145	−0.983	0.192
	SI3	0.70				3.78	1.145	−0.802	−0.189
	SI4	0.77				3.71	1.154	−0.741	−0.217
FC	FC1	0.70	0.83	0.83	0.63	3.30	1.284	−0.401	−0.939
	FC2	0.81				3.67	1.362	−0.752	−0.708
	FC3	0.85				3.75	1.378	−0.865	−0.585
PV	PV1	0.79	0.86	0.87	0.62	3.60	1.136	−0.590	−0.272
	PV2	0.78				3.72	1.133	−0.879	0.141
	PV3	0.83				3.61	1.192	−0.606	−0.444
	PV4	0.74				3.47	1.208	−0.503	−0.593
PR	PR 1	0.67	0.83	0.84	0.56	2.82	1.252	0.201	−1.030
	PR2	0.72				2.25	1.128	0.892	0.078
	PR 3	0.79				2.39	1.142	0.627	−0.394
	PR 4	0.81				2.60	1.164	0.366	−0.678
PT	PT1	0.84	0.86	0.86	0.67	3.29	1.137	−0.373	−0.603
	PT2	0.87				3.43	1.116	−0.460	−0.503
	PT3	0.74				3.36	1.080	−0.266	−0.550
BI	BI1	0.82	0.90	0.90	0.69	3.53	1.246	−0.817	−0.343
	BI2	0.85				3.49	1.238	−0.780	−0.447
	BI3	0.83				3.60	1.259	−0.820	−0.359
	BI4	0.81				3.37	1.274	−0.577	−0.712
UB	UB1	0.76	0.83	0.83	0.62	3.64	1.134	−0.767	−0.279
	UB2	0.83				3.46	1.120	−0.503	−0.528
	UB3	0.78				3.41	1.174	−0.380	−0.796

**Table 8:** Fitting results of the SEM.

Result	χ^2^/df	RMSEA	GFI	AGFI	NFI	IFI	TLI	CFI
Recommended value	<3.0	<0.05	>0.9	>0.9	>0.9	>0.9	>0.9	>0.9
Actual value	1.68	0.04	0.89	0.87	0.89	0.95	0.94	0.95
Model fit	Ideal	Ideal	Acceptable	Acceptable	Acceptable	Ideal	Ideal	Ideal

**Table 9:** Results of the path analysis.

Factor	→	Factor	Path Coefficient	S.E.	C.R.
PE	→	Use intention	0.40 ***	0.06	6.72
EE	→	Use intention	0.40 ***	0.06	6.91
SI	→	Use intention	0.14 *	0.05	2.58
FC	→	Use intention	0.15 ***	0.04	3.94
PV	→	Use intention	0.01	0.05	0.28
PR	→	Use intention	−0.31 ***	0.05	−6.11
PT	→	Use intention	0.31 ***	0.06	5.25
Use intention	→	UB	0.48 ***	0.05	8.82

* *p* < 0.05, *** *p* < 0.001.

## Data Availability

Not applicable.
